# Supplementary material for: Deletion of wfs1 Impairs Oligodendrocyte Precursor Cells Dorsal Distribution and Myelination Through the wfs1-hmgcs1 Axis in Zebrafish
Source: Biology (Basel). 2026 Aug 16;15(16):1402. doi: 10.3390/biology15161402 (PMC13510151; doi:10.3390/biology15161402)
Supplement: Supplementary file 1 [file biology-15-01402-s001.zip › File S1-Transcriptome sequencing data and differentially expressed genes (DEGs) in wfs1-- zebrafish at 5 dpf/2.KEGG/wfs1KOvsWT/DOWN/src/dre03250.html]

dre03250


- dre:562808

- Down regulated genes

562808\_1(-1.27706175077607)
- dre:559032

- Down regulated genes

559032\_1(-1.36919788133913)

- dre:559677

- Down regulated genes

559677\_1(-6.60324462452163)

- dre:321602

- Down regulated genes

321602\_1(-2.43796318075763)

Close
